# SARS-CoV-2 Fusion Peptide Conjugated to a Tetravalent Dendrimer Selectively Inhibits Viral Infection

**DOI:** 10.3390/pharmaceutics15122791

**Published:** 2023-12-17

**Authors:** Carla Zannella, Annalisa Chianese, Alessandra Monti, Rosa Giugliano, Maria Vittoria Morone, Francesco Secci, Giuseppina Sanna, Aldo Manzin, Anna De Filippis, Nunzianna Doti, Massimiliano Galdiero

**Affiliations:** 1 Department of Experimental Medicine, University of Campania “Luigi Vanvitelli”, 80138 Naples, Italy; carla.zannella@unicampania.it (C.Z.); annalisa.chianese@unicampania.it (A.C.); rosa.giugliano@unicampania.it (R.G.); mariavittoria.morone@unicampania.it (M.V.M.); anna.defilippis@unicampania.it (A.D.F.); 2 Institute of Biostructures and Bioimaging (IBB), National Research Council (CNR), 80131 Naples, Italy; alessandra.monti@ibb.cnr.it (A.M.); nunzianna.doti@cnr.it (N.D.); 3 Department of Chemical and Geological Sciences, University of Cagliari, University Campus, 09042 Cagliari, Italy; fsecci@unica.it; 4 Department of Biomedical Sciences, University of Cagliari, University Campus, 09042 Cagliari, Italy; g.sanna@unica.it (G.S.); aldo.manzin@unica.it (A.M.); 5 UOC of Virology and Microbiology, University Hospital of Campania “Luigi Vanvitelli”, 80138 Naples, Italy

**Keywords:** dendrimer, fusion, viral fusion proteins, fusion peptide, SARS-CoV-2, spike, inhibitors, peptide

## Abstract

Fusion is a key event for enveloped viruses, through which viral and cell membranes come into close contact. This event is mediated by viral fusion proteins, which are divided into three structural and functional classes. The severe acute respiratory syndrome coronavirus 2 (SARS-CoV-2) spike protein belongs to class I fusion proteins, characterized by a trimer of helical hairpins and an internal fusion peptide (FP), which is exposed once fusion occurs. Many efforts have been directed at finding antivirals capable of interfering with the fusion mechanism, mainly by designing peptides on the two heptad-repeat regions present in class I viral fusion proteins. Here, we aimed to evaluate the anti-SARS-CoV-2 activity of the FP sequence conjugated to a tetravalent dendrimer through a classical organic nucleophilic substitution reaction (S_N_2) using a synthetic bromoacetylated peptide mimicking the FP and a branched scaffold of poly-*L*-Lysine functionalized with cysteine residues. We found that the FP peptide conjugated to the dendrimer, unlike the monomeric FP sequence, has virucidal activity by impairing the attachment of SARS-CoV-2 to cells. Furthermore, we found that the peptide dendrimer does not have the same effects on other coronaviruses, demonstrating that it is selective against SARS-CoV-2.

## 1. Introduction

The worrying boost in viral respiratory infections has increased interest in the search for new therapeutic agents. Many drugs, with multiple mechanisms of action, have emerged during the COVID-19 outbreak, and three of them have been approved for clinical use [1]. Remdesivir, which inhibits the viral RNA-dependent RNA polymerase, is the first drug approved by the FDA against SARS-CoV-2. It is recommended for the treatment of hospitalized and non-hospitalized people with mild or moderate symptoms [2]. Paxlovid is an oral combination consisting of nirmatrelvir, an inhibitor of the SARS-CoV-2 main protease (Mpro), and ritonavir, an inhibitor of the cytochrome P450-3A4. Its use is indicated for non-hospitalized people with mild or moderate COVID-19 [3]. Molnupiravir is the last treatment option in patients who cannot take either remdesivir or Paxlovid. It is a viral replication inhibitor recommended in non-hospitalized adult people [4]. The use of these three drugs is very controversial, and the FDA has remodulated their administration several times, especially due to the appearance of various side effects and resistant strains [5,6,7,8]. 

In recent years, peptides have gained increasing interest as therapeutic agents against infectious diseases [9,10]. Peptide drugs offer several advantages, such as their low ability to induce resistance, high specificity, efficacy, tolerability, and safety, as well as ease of synthesis [11,12]. They can derive from nature as the antimicrobial peptides (AMPs) isolated from mammals, insects, reptiles, amphibians, plants, and fishes [13,14,15,16,17,18,19] or have a synthetic origin via recombinant and chemical methods [20]. However, peptides as drugs have two drawbacks, membrane impermeability and poor in vivo stability, which have greatly impaired their clinical use. To date, several synthetic strategies have been developed to overcome these problems, including modifications of C- and/or N-terminals, the introduction of D- or other unnatural amino acids, modification of the backbone, PEGylation, and cyclization [12,21,22]. Furthermore, in recent years, to significantly improve the pharmacokinetic properties of peptides, dendrimers have been used effectively as carriers of these molecules [23,24,25]. The characteristic branched structure of peptide dendrimers either with a non-peptide (such as poly-amidoamine) or peptide (such as poly-lysine) core has valuable properties, improves stability, and enhances peptide functionality compared to monomeric peptides [26,27]. For example, compared to their monomeric analogues, the well-known “Multiple Antigen Peptides” (MAP) have shown greater biological activity, as a greater local concentration of bioactive units in the polyvalent assemblies and greater stability against peptidases and proteases have been observed [28]. MAPs, first introduced by Tam in 1988, are dendron-like molecular constructs based on a poly-lysine skeleton, and have found several biological applications, e.g., vaccines and diagnostic research [28]. 

MAPs have also been described as antimicrobial agents acting primarily on bacterial targets [29,30]. For instance, SB056 is a dimeric dendrimer characterized by a core of lysine and two copies of a 10-mer peptide (WKKIRVRLSA) conjugated to an octanamide tail. It is active against some Gram-positive and a wide variety of Gram-negative bacteria, and it is also endowed with antibiofilm properties [28,31]. To date, less is known about the use of peptide dendrimers as antivirals [32,33,34]. Donalisio et al. described two peptide dendrimers, named SB105 and its derivative SB105-A10. The two compounds were able to inhibit genital infection caused by high-risk human papillomaviruses (HPVs) (HPV16, HPV18, and HPV6) in vitro [35], by interacting with heparin and with heparan sulfates exposed on the cell surface, thus interfering with the virus attachment. Furthermore, they also showed a strong antiviral effect against human herpesviruses by blocking the binding to host cells [36,37].

Here, we tested the biological activity of a peptide dendrimer against severe acute respiratory syndrome coronavirus 2 (SARS-CoV-2). The Spike (S) protein finely tuned the entry process of SARS-CoV-2. The S protein consists of two subunits: S1, responsible for recognizing the cellular receptor, i.e., human angiotensin-converting enzyme 2 (ACE2), and S2, essential for the virus–host membrane fusion. Similar to what has been discovered for other viruses, such as influenza hemagglutinin (HA), human immunodeficiency virus (HIV) Env, and Ebola GP, the fusion process of SARS-CoV-2 with the host cell requires some steps starting from the interaction between the S1 receptor and ACE2. Then, several cleavages occur in S2 downstream of the two heptad (HR1 and HR2) repeat regions, dissociating S1 from the S protein and leading to a series of conformational changes that culminate in the exposure and fusion of the fusion peptide (FP) into the host cell. Numerous studies have reported the antiviral activity of peptides designed on HR1 and HR2 of different viral surface glycoproteins [38,39,40,41,42,43,44], but little is known about fusion peptide inhibitors.

In this framework, we report the synthesis and characterization of a tetravalent dendrimer conjugated to the FP sequence of SARS-CoV-2 (^788^IYKTPPIDFGGFNFSQIL^806^). The synthesis protocol involves the derivatization of a poly-lysine core with cysteine residues on its Nα and NƐ positions and its conjugation in solution with a bromoacetylated peptide through a thioether bond formation [45,46]. The antiviral activity of the peptide dendrimer (hereafter, dendrimer R1) was evaluated against SARS-CoV-2 infection and compared to that of the monomeric linear synthetic FP peptide (R1) and the dendrimer core (dendrimer). The results showed that dendrimer R1 has marked anti-SARS-CoV-2 activity preventing its initial attachment phase to the host cell. In addition, this activity is specifically directed to SARS-CoV-2 and not to other coronaviruses.

## 2. Materials and Methods

### 2.1. Reagents

All chemicals were acquired from commercial sources and used without further purification unless otherwise stated. Solvents and reagents, including acetonitrile (CH_3_CN), dimethylformamide (DMF), sym-collidine, N,N-diisopropylethylamine (DIPEA), piperidine, acetic anhydride (Ac2O), trifluoroacetic acid (TFA), and Tris (2-carboxyethyl) phosphine (TCEP) were purchased from Sigma-Aldrich (Milan, Italy). Protected amino acids and coupling agents, such as 1-[Bis(dimethylamino)methylene]-1H-1,2,3-triazolo [4,5-b]pyridinium 3-oxid hexafluorophosphate (HATU), ethyl 2-cyano-2-(hydroxyimino)acetate (OxymaPure^®^, Marktrewitz, Germany), and N-N’ diisopropylcarbodiimide (DIC) used for peptide synthesis, were acquired from IRIS Biotech GmbH.

### 2.2. Peptide and Branched Amino Acid Core Synthesis and Characterization

The FP mimetic peptide (Ile-Tyr-Lys-Thr-Pro-Pro-Ile-Lys-Asp-Phe-Gly-Gly-Phe-Asn-Phe-Ser-Gln-Ile-Leu; GenBank: QHD43416) was synthesized as an amidated derivative at the C-terminus using Rink-Amide MBHA resin (loading 0.5 mmol/g) following the N-9-Fluorenylmethyloxycarbonyl (Fmoc) strategy, utilizing a SYRO I peptide synthesizer, as reported elsewhere [47]. As the final step of the synthesis, bromoacetic acid was reacted with the peptide amino-terminus to form the bromoacetyl-derivatized fully protected peptide (Br-CH_2_CO-Ile-Tyr-Lys-Thr-Pro-Pro-Ile-Lys-Asp-Phe-Gly-Gly-Phe-Asn-Phe-Ser-Gln-Ile-Leu-NH_2_) (Scheme S1 in the Supplementary Materials). Bromoacetylation was performed using a 10-fold excess of bromoacetic acid and DIC as activators (1:1 eq.) in DMF for 1 h at room temperature.

The tetrabranched core ((Cys)_4_-(Lys)_2_-Lys-Ala-NH_2_) was manually prepared starting from a C-terminal alanine and by incorporating the lysine residues in two sequential couplings and deprotection cycles with Fmoc-L-Lys(Fmoc)-OH, using Rink-Amide MBHA resin (0.5 mmol/g loading). Amino acids were used in 4-fold excess in DMF, utilizing HATU/sim-collidine (1:2 eq.) as activating reagents and piperidine for Fmoc removal. As depicted in Scheme S1 in the Supplementary Materials, the process led to a resin-anchored branched trilysine core provided with four amino groups (two α-amino and two ε-amino groups) to which cysteine residues were coupled to achieve the final structure (Cys)_4_-(Lys)_2_-Lys-Ala-NH_2_.

The bromoacetylated peptide and the branched core were cleaved from the resin and freed of the side chain’s protective groups by treatment with a TFA/triisopropylsilane (TIS)/water (95: 2.5: 2.5 *v*/*v*/*v*) mixture, under stirring, for 3 h at room temperature. Peptides were precipitated with cold diethyl ether, extracted with a mixture of H_2_O/CH_3_CN (75:25 *v*/*v*), and freeze-dried. The identity and purity of the peptides were assessed by liquid chromatography–mass spectrometry (LC-MS) using an Agilent 1290 Infinity ESI-TOF-MS LC coupled to an Agilent 6230 time-of-flight LC/MS system (TOF) (Agilent Technologies, Cernusco sul Naviglio, Italy) with a Waters xBridge C18 column (3 μm, 4.6 × 5.0 mm), applying a linear gradient of CH3CN/0.05% TFA in water with 0.05% TFA from 5 to 70% for 15 min, at a flow rate of 0.2 mL/min. Raw peptides with a degree of purity greater than 90% were used for assembling the peptide dendrimer.

### 2.3. Conjugation Reaction, Purification, and Characterization of the Peptide Dendrimer

**Figure 1:**
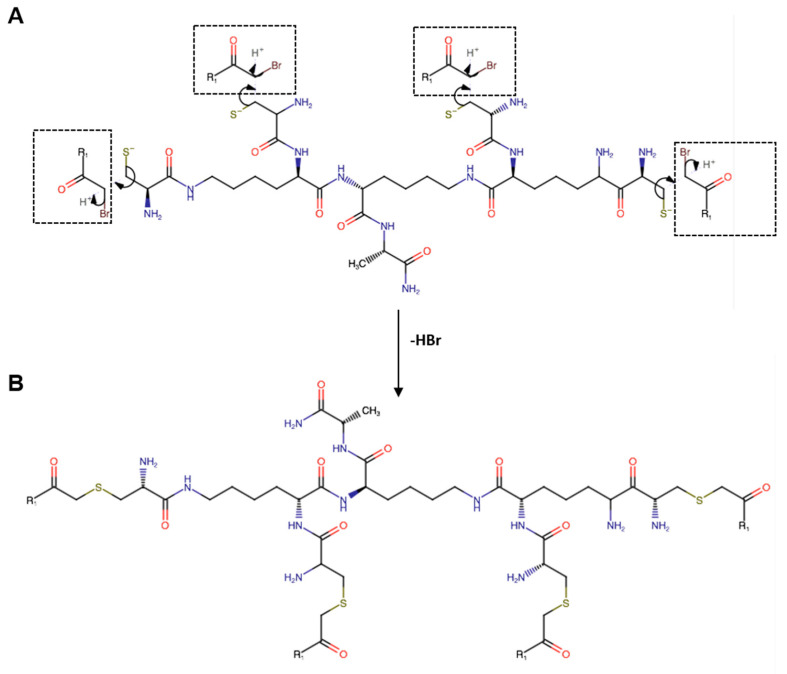
(**A**) Schematic representation of the site-specific nucleophilic substitution reaction (SN2) between the bromoacetylated monomeric peptide R1 (Br-CH_2_CO-R1) and the thiol (-SH) group of cysteine residues of the branched amino acid core. (**B**) Chemical structure of the peptide dendrimer R1. All the amino acids have an L configuration.

The FP peptide was conjugated to the four dendrimers’ thiol groups according to the organic nucleophilic substitution reaction (SN2) (Figure 1). (Cys)_4_-(Lys)_2_-Lys-Ala-NH_2_ was first treated with TCEP at 0.4 mM for 30 min in water, and then, diluted to a final concentration of 0.5 mg/mL in a solution of CH_3_CN/NH_4_HCO_3_ 40 mM (25:75 *v*/*v*) at pH 8.0. Then, a 5-fold excess, with respect to the thiol groups, of the FP peptide was added drop-wise for 16 h at room temperature. The final product was purified by reversed phase HPLC (RP-HPLC) on a WATERS 2545 preparative system (Waters, Milan, Italy) equipped with a WATERS 2489 UV/Vis detector. The purification was performed at 15 mL/min using a Jupiter C18 column (5 μm, 150 × 21.2 mm ID) applying a linear gradient of 0.1% TFA in CH_3_CN from 5% to 70% for 30 min, monitoring the absorbance at 210 nm. The relative purity of peptides was calculated as the ratio of the peak area of the target peptide and the sum of areas of all detected peaks from the UV chromatograms at 210 nm. The purity of all peptides was >95%. Peptide concentration was determined by reading the absorbance at 280 nm using a NanoDrop2000c UV-Vis spectrophotometer (Thermo Scientific, Waltham, MA, USA).

### 2.4. Cells and Viruses

*Cercopithecus aethiops* kidney cells (Vero-76, ATCC CRL 1587) and human lung adenocarcinoma Calu-3 (ATCC HTB-55) were purchased from the American Type Culture Collection (ATCC, Manassas, VA, USA). Vero-76 cells were cultured in Dulbecco’s Modified Eagle’s Medium (DMEM, Microtech, Naples, Italy) supplemented with 10% Fetal Bovine Serum (FBS, Microgem, Naples, Italy) and antibiotic solution (10,000 U penicillin + 10 mg streptomycin, Himedia, Mumbai, India). Calu-3 cells were grown in Eagle’s Minimum Essential Medium (EMEM, Microtech) supplemented with 10% FBS antibiotic solution. All the coronaviruses used in this study, which are (i) HCoV-229E (ATCC VR-740); (ii) HCoV-OC43 (ATCC VR-1558); and (iii) SARS-CoV-2 (clinical isolate, strain VR PV10734, kindly donated by the Lazzaro Spallanzani Hospital, Rome, Italy), were propagated in the Vero-76 cell line and their concentration was determined via plaque assay [20]. For HCoV-OC43, the viral concentration was expressed as the median tissue culture infectious dose (TCID_50_). All experimental work involving SARS-CoV-2 was performed in a biosafety level (BSL) 3 laboratory.

### 2.5. Cell Viability Assay

Vero-76 cells were seeded in 96-well plates at a density of 2 × 10^4^ cells/well. The day after, cells were treated with different concentrations of each compound (R1, dendrimer and dendrimer R1) from 5 to 100 μM, and incubated for 24 h at 37 °C and 5% CO_2_. Cell viability was evaluated via a 3-(4,5-dimethylthiazol-2-yl)-2,5-diphenyltetrazolium bromide (MTT) assay, and it corresponds to:

[1 − (Abs of treated samples − Abs of blank/Abs of control samples − Abs of blank)] × 100,

where Abs of blank and control samples refer to the absorbance of solvent and not treated cells (indicated in the figures as CTRL+), respectively. Negative control (indicated in the figures as CTRL−) refers to cells treated with 100% dimethyl-sulfoxide (DMSO). The 50% cytotoxicity concentration (CC_50_) was calculated via linear regression analysis.

### 2.6. Antiviral Assays: HCoV-229E and SARS-CoV-2

The antiviral activity of compounds was analyzed through four different schemes of treatment as previously reported [20,48,49]. Vero-76 cells were seeded in 24-well plates (1.2 × 10^5^ cells/well) and grown for 24 h at 37 °C in 5% CO_2_. In all assays, performed in triplicate, compounds were added to the medium without FBS at concentrations of 50, 20, 10, and 5 μM. The inhibition rate of viral infectivity was calculated by comparing the number of plaques obtained in cells treated with each compound to the plaques counted in the CTRL− (cells infected with virus, without any compound). The 50% inhibitory concentration (IC_50_) was calculated via linear regression analysis.

#### 2.6.1. Co-Treatment Assay

Vero-76 cells were simultaneously incubated with each compound and virus at a multiplicity of infection (MOI) of 0.01 pfu/cell for 2 h at 37 °C. Then, the mixture (compound + virus) was removed from cells; the Vero-76 monolayer was washed three times with citrate buffer (pH 3.0) for 5 min, then overlaid with the complete medium (10% FBS), supplemented with carboxymethylcellulose (CMC) at 5% (Sigma, C5678, C5013), and finally incubated for 48 h at 37 °C and 5% CO_2_. Finally, the monolayers were fixed with formaldehyde (Sigma-Aldrich, St. Louis, MO, USA) 4% and stained with crystal violet solution (Sigma-Aldrich), and the plaques were counted.

#### 2.6.2. Cell Pre-Treatment Assay

Vero-76 cells were first treated with each compound for 1 h. Then, each virus was added to a MOI of 0.01 pfu/mL for 2 h at 37 °C. After that, they were removed, and cells were washed with citrate buffer and supplemented with CMC for 48 h at 37 °C. At the end, the cell monolayer was fixed and stained, and plaques were scored.

#### 2.6.3. Virucidal Assay

Each compound and virus (1 × 10^4^ pfu/mL) was incubated for 1 h at 37 °C. After that, each mixture (compound + virus) was diluted so that the compound reached a nonactive concentration and the virus was at an MOI of 0.01 pfu/cell. Dilutions were added to Vero-76 monolayer for 2 h, and then, the cells were washed with citrate buffer and overlaid with CMC for 48 h. Finally, the cells were fixed and stained, and the number of plaques counted.

#### 2.6.4. Post-Treatment Assay

Vero-76 cells were first infected with the virus (MOI 0.01) for 2 h at 37 °C; then, the cell monolayer was washed with citrate buffer and incubated with each compound in the presence of CMC for 48 h at 37 °C. Cells were then fixed and stained, and the viral plaques scored.

### 2.7. Antiviral Assays: HCoV-OC43

This test was based on the inhibition of virus-induced cytopathogenicity in the Vero-76 cell monolayer [20]. In detail, cells were seeded in 96-well plates (2 × 10^4^ cells/well) and grown for 24 h at 37 °C in 5% CO_2_. In all the assays, performed in triplicate, compounds were added to the medium in the presence of 5% FBS at noncytotoxic concentrations of 50, 20, 10, and 5 μM. (i) Co-treatment assay: cells were incubated with each compound and infected with HCoV-OC43 (200 TCID_50_/mL) at the same time (2 h at 37 °C); (ii) cell pre-treatment assay: cells were previously treated with compound, and then, infected (200 TCID_50_/mL; 2 h at 37 °C); (iii) virucidal assay: each compound was incubated together with the virus during the adsorption step, and then, mixture was diluted on Vero-76 cells (2 h at 37 °C); (iv) post-treatment assay: each compound was added to cells after the viral adsorption step (2 h at 37 °C). At the end of each assay, culture medium to which we added 5% FBS was incubated on cells and, after a 5-day incubation at 37 °C, a cytopathic effect (CPE) was observed. Cells were stained with MTT solution as reported in Section 2.4. The inhibition rate of viral infectivity was calculated as follows:

[1 − (Abs of treated samples − Abs of blank/Abs of control samples − Abs of blank)] × 100,

where Abs of blank and control samples refer to the absorbance of non-treated cells (CTRL+) and infected cells (CTRL−), respectively.

### 2.8. Temperature-Shift Assays: SARS-CoV-2

(i) Entry assay: Pre-cooled Vero-76 cells were first infected with SARS-CoV-2 (MOI 0.01) for 2 h at 4 °C to permit viral attachment. Then, cells were washed three times with citrate buffer (pH 3) and treated with compounds or heparin for 1 h at 37 °C. After the treatment period, cells were coated with CMC to which we added a culture medium, and incubated for 48 h at 37 °C. Vero-76 cells were finally fixed and stained with crystal violet, and the viral plaques were counted as previously reported [13]. (ii) Attachment assay: Pre-cooled Vero-76 cell monolayers were infected with SARS-CoV-2 (MOI 0.01) in the presence of the compounds or heparin 10 μM (CTRL+) for 2 h at 4 °C. Subsequently, cells were washed with citrate buffer (pH 3) to remove unabsorbed viruses, overlaid with CMC supplemented with culture medium, and incubated for 48 h at 37 °C. Finally, cells were fixed and stained with crystal violet and viral plaques were counted under the microscope [13].

### 2.9. Yield Reduction Assay

A virucidal assay was conducted as described in Section 2.6 and [50]. In brief, each compound was incubated together with the virus (200 TCID_50_/mL) during the adsorption step, and then, the mixture was diluted on Calu-3 cells (5 × 10^5^/mL). After 96 h at 37 °C and 5% CO_2_, each sample was harvested and stored at −80 °C. Samples were then diluted with serial passages, starting from 10^−1^ and increasing to 10^−10^. The titer of the virus-containing supernatant dilution series was determined by the TCID_50_/mL end-point in Vero-76.

### 2.10. Statistical Analysis

All experiments were performed in triplicate and are expressed as mean ± standard deviation (SD), with calculations performed using GraphPad Prism version 5 (La Jolla, CA, USA). Statistical differences were evaluated via one-way ANOVA followed by Dunnett’s multiple comparisons test, and a value of *p* ≤ 0.05 was considered significant.

### 2.11. Molecular Docking

The S protein structures (SARS-CoV-2, PDB 6XM4; HCoV-OC43, PDB 7SBW; HCoV-229E, PDB 7CYC) were obtained from the protein database. The chemical and the secondary structures of SARS-CoV-2 FP were modeled via ChemBio3D Ultra 13.0. (Colorado Springs, Colorado, USA). Molecular docking was carried out by using the HPEPDOCK web server with its default parameters. We added the structure of S protein as the receptor input, and HPEPDOCK generated tridimensional structure models for the peptide sequence using the implemented MODPEP program [20]. In addition, we analyzed the interactions between the S protein and the FP via PyMOL software and solved them using iGEMDOCK v2.1 software (Hsinchu, Taiwan).

## 3. Results

### 3.1. Design and Preparation of Dendrimer R1

The aim of the present study was to evaluate the antiviral activity of the SARS-CoV-2 FP sequence conjugated to a tetravalent dendrimer (dendrimer R1). The SARS-CoV-2 S2 subunit is deputed to fusion, similarly to other class I fusion proteins. It consists of some critical regions, i.e., two HRs, namely HR1 and HR2, upstream of the transmembrane (TM) domain, and the FP [47,51,52]. HR1 and HR2 are highly conserved among coronaviruses and therefore are important targets for the development of fusion inhibitors. On the contrary, no inhibitor targeting the SARS-CoV-2 FP has been described until now, probably because the FP sequence diverged during the evolution of coronaviruses.

Dendrimer R1 (Figure 1B) was synthesized by a site-specific nucleophilic substitution reaction 2 (SN2) between the bromoacetylated peptide R1 (Br-CH_2_CO-Ile-Tyr-Lys-Thr-Pro-Pro-Ile-Lys-Asp-Phe-Gly-Gly-Phe-Asn-Phe-Ser-Gln-Ile-Leu-NH_2_) and the thiol group (-SH) of cysteine residues of the branched poly-Lys amino acid core ((Cys)_4_-(Lys)_2_-Lys-Ala-NH_2_). The monomeric peptide and the branched amino acid core were synthesized using the standard solid-phase-Fmoc method, as detailed in the Materials and Methods section. Under our analytical conditions, the retention time (tR) for the desired monomeric peptide R1 (Supplementary Figure S1A) was 13.91 min (Supplementary Figure S1B,C); MS analysis showed the expected mass at *m*/*z*: 1153.582 ([M+2H]^2+^) and 769.391 ([M+3H]^3+^) (Supplementary Figure S1D). The tR of the branched amino acid core (Supplementary Figure S2A) was 6.77 min (Supplementary Figures S1B and S2C), and MS analysis (Supplementary Figure S2D) confirmed the identity of the molecule showing m/z values at 907.392 ([M+Na]^+^); 885.401 ([M+H]^+^); and 443.209 ([M+2H]^2+^). Finally, dendrimer R1 (Supplementary Figure S3A) was eluted at tR 9.07 min (Supplementary Figure S3B,C), and the experimental MW was at *m*/*z* 2446.827 ([M+4H]^4+^), 1957.662 ([M+5H]^5+^), and 1631.597 ([M+6H]^6+^). The deconvolute mass of 9782.82 amu (D) agrees with the theoretical MW of the molecule. The yield of the final product was on average above 50%, and the purity above 95%. The experimental and theoretical MWs of the molecules are shown in Supplementary Table S1.

### 3.2. Cytotoxicity Analysis

**Figure 2:**
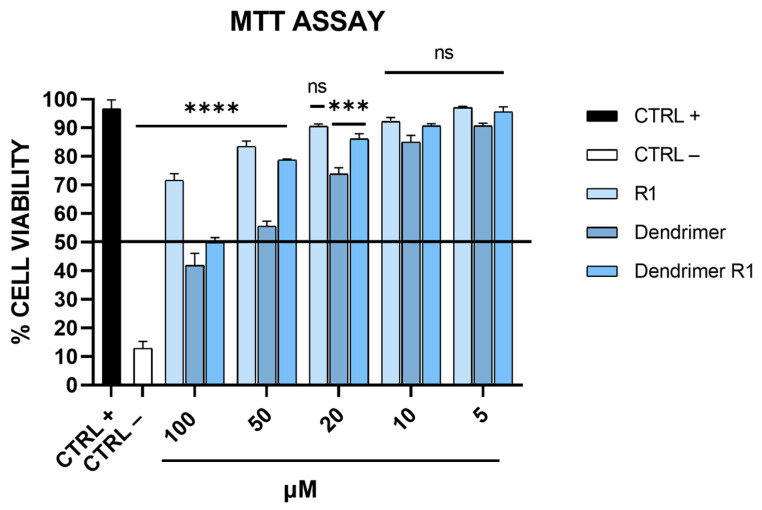
Toxicity evaluation on Vero-76 cells. Cell monolayers were treated with compounds at different concentrations (5, 10, 20, 50, and 100 μM). After 24 h, cell viability was determined via MTT assay. CTRL+ refers to untreated cells, and CTRL− indicates DMSO-treated cells. **** *p* < 0.0001; *** *p* = 0.003; ns: not significant.

The toxicity levels of peptide R1, the branched amino acid core (dendrimer), and the peptide dendrimer (dendrimer R1) were evaluated on Vero-76 cell monolayers by the MTT assay (Figure 2).

As shown in Figure 2, the MTT assay revealed that the compounds have a dose-dependent cytotoxic effect on Vero-76 cells. In particular, R1 does not show toxicity at the tested concentrations; meanwhile, the dendrimer is toxic at concentrations ranging from 100 to 50 μM. Dendrimer R1 does not significantly reduce the viability of Vero-76 cells after 24 h of incubation, except for the concentration of 100 μM. Based on these data, we set the experimental concentration range for the subsequent assays from 5 to 50 μM.

### 3.3. Antiviral Activity against Vero-76 Cells

**Figure 3:**
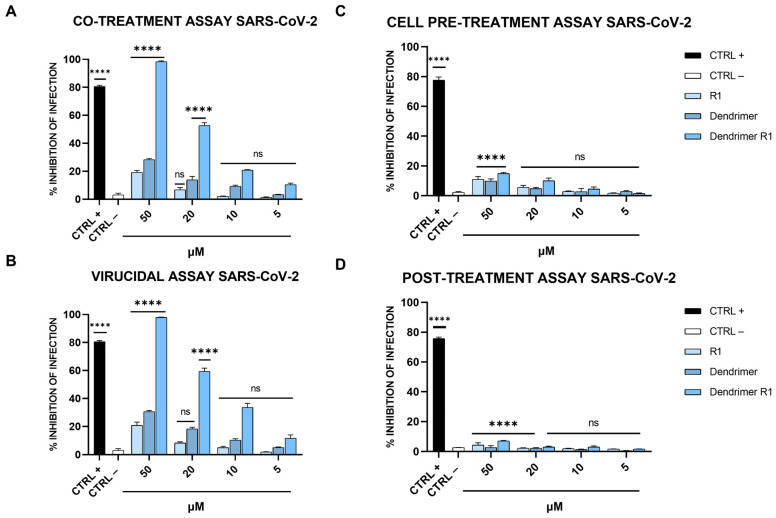
Anti-SARS-CoV-2 activity. (**A**) Co-treatment assay; (**B**) virucidal assay; (**C**) cell pre-treatment assay; and (**D**) post-treatment assay. Cells were first infected, and then, treated with the compound. Cells were infected and treated with ivermectin [20] at 12 μM were used as an internal control and reported as CTRL+, while CTRL− refers to infected cells. The data shown in each column are the means ± standard deviations (SD; error bars) from three independent experiments performed in duplicate. **** *p* < 0.0001; ns: not significant.

The antiviral activity of compounds (R1, dendrimer, and dendrimer R1) against SARS-CoV-2 infection was assessed via plaque assays (Figure 3). In detail, we performed four different experiments: (i) co-treatment assays, in which the cells, the virus, and each compound are incubated together; (ii) virucidal assays, in which the compounds are first incubated with the virus, and then, the mixture is added to the cells; (iii) cell pretreatment assays, in which the compounds are first incubated with the cells, and then, the virus is added; and (iv) post-treatment assays, in which the cells are first infected, and then, treated with the compounds (for details, see Materials and Methods section). In Figure 3A, the results obtained in the co-treatment experiment are shown.

The data obtained showed that R1 and the dendrimer have no inhibitory effect against SARS-CoV-2 in co-treatment experiments. In fact, the percentage of infection inhibition reaches only 15–20% at the maximum concentration tested (50 µM). In contrast, dendrimer R1 shows marked dose-dependent antiviral activity with an IC_50_ value of 20 μM. In the virucidal assay (Figure 3B), both R1 and the dendrimer are inactive, while dendrimer R1 provides antiviral activity, showing an IC_50_ value of 15 μM. In the cell pre-treatment and post-treatment assays, none of the compounds in the concentration range used show inhibitory activity against SARS-CoV-2 replication (Figure 3C,D). The lack of activity of dendrimer R1 is probably due to the fact that this construct is able to penetrate inside the cell in line with its conjugation with the membranotropic FP sequence. Therefore, during the 1 h of cell pretreatment, the majority of dendrimer R1 may have already translocated inside the cell, where it is inactive according to the post-treatment assay (Figure 3C,D). The amount of dendrimer R1 left on the cell surface may be below the concentration needed to maintain antiviral activity.

The relative effectiveness of dendrimer R1 in inhibiting viral replication can be compared to cell viability (CC_50_ value/IC_50_ value) to obtain the therapeutic index (TI), which corresponds to 5 and 6.6 in the co-treatment and virucidal assays, respectively. On the contrary, the TI is unable to calculate for the monomeric parts, i.e., R1 and the dendrimer, whose inhibition of infection do not reach the IC_50_. Taken together, these data indicate that dendrimer R1 has antiviral activity by acting directly on the viral particles. Conjugation with the dendrimer could improve its targeting toward the S protein exposed on the viral envelope and the subsequent interference with the fusion event.

**Figure 4:**
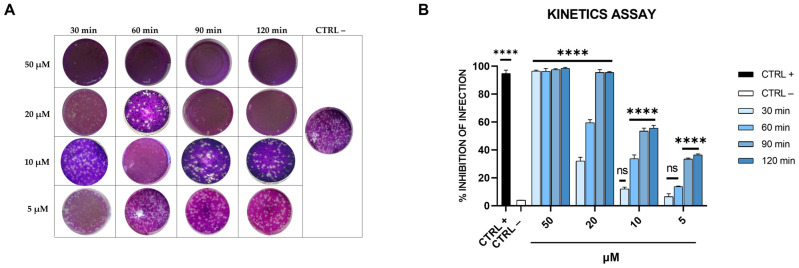
Kinetics assay. (**A**) Representative plaques of SARS-CoV-2 on Vero-76 cells stained with crystal violet. Dendrimer R1 was incubated with SARS-CoV-2 for 30, 60, 90, and 120 min at different concentrations (5, 10, 20, and 50 µM). Therefore, untreated and treated virus suspensions were added to Vero-76 cells. After further incubation, cells were fixed and stained for visualization of viral plaques. Virus-infected cells are indicated as CTRL− and used as internal control. (**B**) Quantitative analysis of plaque reduction in virucidal assay at different virus + compound incubation times and at different compound concentrations. CTRL+ refers to cells infected and treated with ivermectin at 12 μM [20]. All values represent the means ± standard deviations (SD; error bars) of three independent experiments performed in duplicate. **** *p* < 0.0001; ns: not significant.

To further evaluate the effectiveness of dendrimer R1 in inhibiting SARS-CoV-2 infection, we performed a virucidal assay by varying the incubation time of the compound with the virus. In particular, dendrimer R1 was incubated with the virus for 30, 60, 90, and 120 min, and after, the resulting mixtures were diluted onto Vero-76 cells, and plaque assays were performed (Figure 4).

As shown in Figure 4, dendrimer R1 at 50 µM completely blocks the SARS-CoV-2 infection already after 30 min of incubation with the virus. Moreover, by increasing the incubation time, dendrimer R1 significantly interferes with SARS-CoV-2 infection even at the lowest concentrations tested. In particular, at the incubation times of 90 and 120 min, we observed a significant decrease in the infection rate with an IC50 of 10 μM.

**Figure 5:**
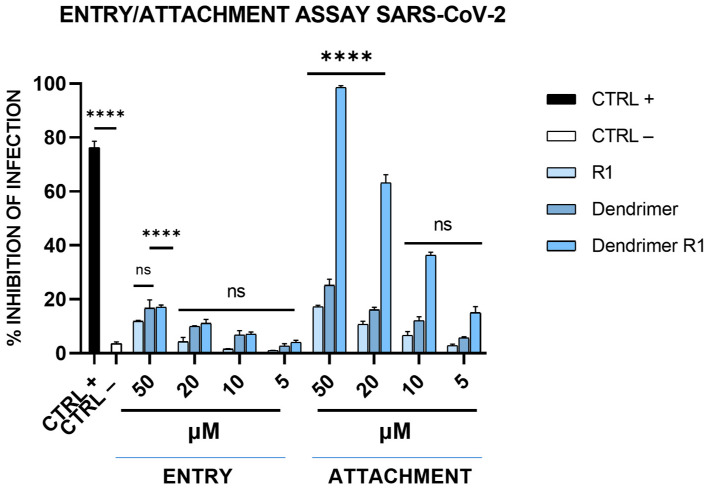
SARS-CoV-2 entry and attachment tests. In the entry assay, Vero-76 cells were previously infected at 4 °C, and then, the compound incubation was shifted at 37 °C. In the attachment assay, cells were simultaneously incubated with each compound and virus at 4 °C, and then, the infection proceeded at 37 °C. CTRL+ refers to virus-infected cells treated with heparin at 10 μM [13], and CTRL− indicates virus-infected cells. The data shown in each column are the means ± standard deviations (SD; error bars) from three independent experiments performed in duplicate. **** *p* < 0.0001; ns: not significant.

We also assessed whether dendrimer R1 is an inhibitor of the virus attachment phase or an inhibitor of the virus entry phase (see the Materials and Methods section for details). As shown in Figure 5, dendrimer R1, does not influence viral entry at any of the concentrations tested, while it influences SARS-CoV-2 attachment in a dose-dependent manner and to a similar extent to that observed in the virucidal assay (Figure 3B). The R1 peptide and the dendrimer, however, are inactive in both assays at all concentrations, in line with the antiviral assays previously described. Overall, the data confirm that the antiviral effect of dendrimer R1 is based on its ability to prevent the attachment of SARS-CoV-2 to target cells.

**Figure 6:**
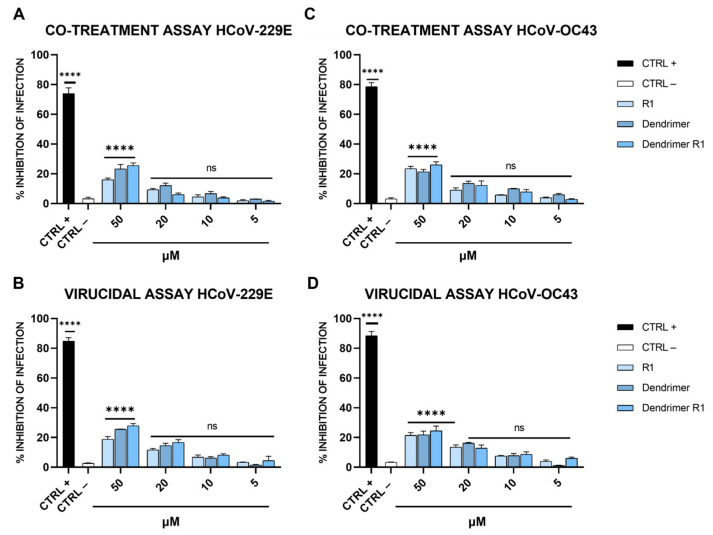
Anti-HCoV-229E and anti-HCoV-OC43 activity. (**A**) Co-treatment and (**B**) virucidal assays against HCoV-229E; (**C**) co-treatment and (**D**) virucidal assays against HCoV-OC43. CTRL+ refers to cells treated with ivermectin at 12 μM [20], and CTRL− indicates virus-infected cells. The data shown in each column are the means ± standard deviations (SD; error bars) from three independent experiments performed in duplicate. **** *p* < 0.0001; ns: not significant.

Finally, to test whether dendrimer R1 could also be active against other coronaviruses, its antiviral potential was further investigated against HCoV-229E and HCoV-OC43 (Figure 6).

As shown in Figure 6, none of the compounds affected SARS-CoV-2 infection at the concentrations examined, indicating that the dendrimer conjugated with the SARS-CoV-2 FP was highly specific. This evidence is not very surprising since coronaviruses differ consistently in their primary sequences and mainly in the FP sequence, even if they maintain very similar essential biological function [53]. In detail, Ou et al. demonstrated that the FPs of different coronaviruses, including HCoV-229E and HCoV-OC43, varied significantly in length and primary sequence, even if they shared a common hydrophobic character and adopted a conserved helix structure in the presence of trifluoroethanol (TFE) [53]. In fact, the FP of SARS-CoV-2 used to modify the dendrimer structure has low homology in the primary sequence compared to those of the coronaviruses tested.

### 3.4. Antiviral Activity on Calu-3 Cells

**Figure 7:**
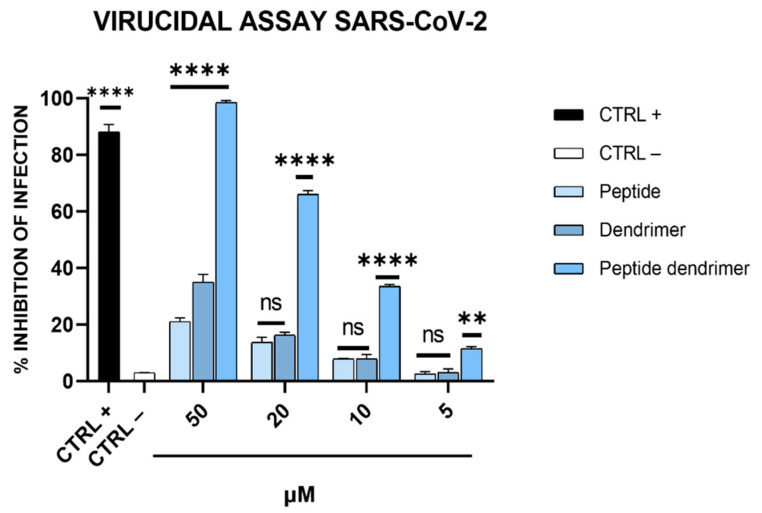
Anti-SARS-CoV-2 activity on Calu-3 cells. Each compound at different concentrations was incubated with the virus (200 TCID50/mL) during the adsorption step, and then, the resulting mixtures were diluted on Calu-3 cells. After 96 h post-infection, each sample was harvested and diluted at the TCID50/mL end-point on Vero-76. CTRL+ refers to virus-infected cells treated with ivermectin at 12 μM [20], while CTRL− indicates virus-infected cells. **** *p* < 0.0001; ** *p* < 0.0020; ns: not significant.

The effectiveness of dendrimer R1 as a virucidal agent was also tested on human cells. Therefore, a virucidal assay was performed on Calu-3 cells as described in Section 2.8. As shown in Figure 7, in line with the results obtained on Vero-76 cells (Figure 3B), dendrimer R1 inhibits SARS-CoV-2 infection in a dose-dependent manner, showing an IC_50_ of 15 μM. Peptide R1 and the dendrimer used as internal controls are ineffective.

### 3.5. In Silico Analysis

**Figure 8:**
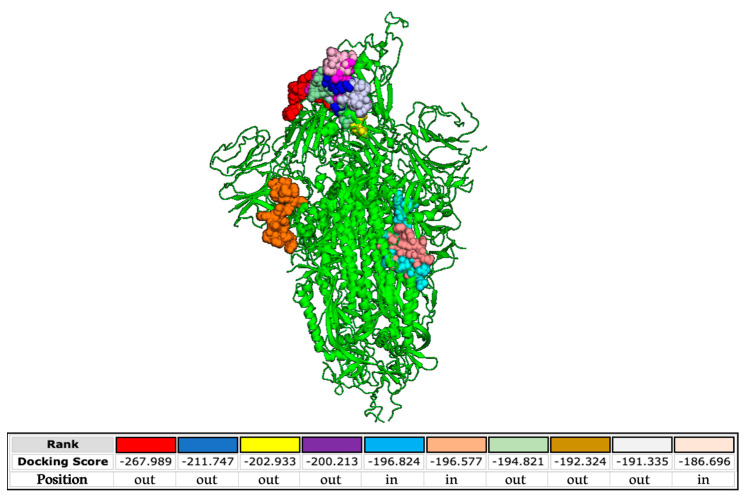
Molecular simulation of SARS-CoV-2 FP interacting with the S protein (PDB 6XM4) obtained by the HPEPDOCK server. The different color codes of the peptide, represented as balls, refer to the different binding free energy.

Finally, we analyzed the interactions occurring between the SARS-CoV-2 S protein and the FP used to engineer the poly(L-lysine) backbone. The docking predictions shown in Figure 8 demonstrate that the FP is able to bind the S protein at different sites present both in the external and inner regions of the SARS-CoV-2 glycoprotein.

The binding free energies are very high, evidencing highly possible interactions. On the contrary, binding between the FP and HCoV-OC43 S protein is not properly probable, as indicated by the lower energy scores (Supplementary Figure S4A). We also investigated the potential interactions between the FP and the alphacoronavirus HCoV-229E (Supplementary Figure S4B). In this case, all the binding sites are in the inner regions of the glycoprotein. These data confirm the absence of activity of the peptide dendrimer when tested against HCoV-229E and HCoV-OC43 (Figure 6) and reinforce its specificity against SARS-CoV-2 infection. In more detail, we predict that the interactions contributing to the binding between the FP and the glycoprotein of SARS-CoV-2 comprise hydrophobic and electrostatic forces (Supplementary Table S2).

## 4. Discussion

The COVID-19 outbreak has revolutionized health, social, and economic systems in the last three years. Even though the World Health Organization (WHO) announced the end of the global health emergence on 5 May 2023, continuous efforts are still aimed at researching antiviral therapies in preparation to face not only COVID-19, but also other new pandemic outbreaks. Here, we present data demonstrating that SARS-COV-2 FP conjugated to a tetravalent dendrimer is capable of interfering with the host–virus fusion process.

The initiation of the viral infectious lifecycle is mediated by viral fusion proteins, which, through the interaction with cell surface molecules or receptors, allow the juxtaposition between virus and cell membranes. This event is coupled with rearrangements of viral fusion proteins, which transit from their pre-fusion form to their energetically favorable post-fusion form. Inhibitors able to prevent this structural refolding offer a great deal of therapeutic and commercial potential since they block the infection prior to the virus’ entry into the host cells. FP inhibitors have not been largely used for interfering with the fusion/entry process with respect to HR-derived inhibitors [44,54,55,56,57,58]. To date, only a few studies have reported FP-targeted peptides as anti-HIV agents [59,60,61]. For instance, Jiang et al. designed a hybrid peptide consisting of an HIV gp41 FP and a gp41 N-terminal HR. The peptide was able to target both the regions of gp41, strongly preventing viral infection [62]. Owens et al. synthesized several oligopeptides modeled on the N-terminal region of gp41 and evaluated their ability to inhibit HIV-induced fusion in CD4^+^ cells [60], identifying a hexapeptide with potent inhibitory properties. Another study described the virus–cell fusion inhibition of the peptide SV-201 derived from a conserved and amphipathic domain within the Sendai virus fusion protein [63]. Lastly, Wu et al. screened a set of peptides designed for influenza A FPs. These peptides are negatively charged, and the authors replaced the negative or neutral amino acid residues in lysine, producing positively charged pseudo-FPs (pFPs) [64]. Their results showed not only that the peptides blocked the infection of influenza A virus, but also that of oseltamivir-resistant strains, by interacting and interfering with the subunit HA2.

Recently, two linked ACE2 fragments were attached to poly-(amidoamine) (PAMAM) dendrimers to block S protein–ACE2 interaction [32]. The obtained peptide dendrimer demonstrated an improved binding affinity of three orders of magnitude compared to the free peptide, as confirmed by surface plasmon resonance. In addition, by using an in vitro assay with SARS-CoV-2-mimicking microbeads, strong inhibition of viral infection was also observed. By moving to other viruses, which expose a class I fusion protein like SARS-CoV-2, a sialic acid-mimic peptide able to bind the influenza virus hemagglutinin (HA) was conjugated to carbosilane dendrimers and tested [65]. The resulting compound strongly reduced the infection caused by A/PR/8/34 (H1N1) and A/Aichi/2/68 (H3N2) influenza viruses. The influenza virus HA has also been targeted by another peptide dendrimer consisting of the HA2 subunit [66]. Apart from analyzing antiviral activity, the authors also studied the levels of NF-κB and proinflammatory cytokines, such as TNF-α, IL-1β, and IL-6, generally associated with influenza infection, which were reduced by the treatment with the HA2 dendrimer. In addition, when the construct was inoculated in mice, it increased the survival rate and reduced the viral load in the lungs. Our group demonstrated the antiviral activity of a peptide dendrimer in the inhibition of Herpes simplex virus (HSV) infection [48]. The molecule is formed of a peptide comprising the HSV glycoprotein H (gH), i.e., gH625-644, critical to its membranotropic nature, and a poly (amide)-based dendrimer. The results showed that the peptide dendrimer was able to interfere with the entry mechanism of both HSV type 1 (HSV-1) and type 2 (HSV-2), probably by interacting with the glycoproteins deputed to the early stages of attachment and entry into the target cell and blocking the juxtaposition of the two membranes.

Here, we showed for the first time that a SARS-CoV-2 FP sequence conjugated to a tetravalent dendrimer (dendrimer R1) shows marked antiviral activity (Figure 3), targeting the initial attachment of the virus to the host cell (Figure 5) already after 30 min of incubation (Figure 4). The effect of dendrimer R1 is restricted only to SARS-CoV-2 (Figure 6) since it is not active against HCoV-229E and HCoV-OC43. Although the mechanism of action of the new compound has not been completely clarified, we hypothesize that the hydrophobic and electrostatic interactions between SARS-CoV-2 FP and proteins on the viral surface may represent the main binding forces that prevent/interfere with rearrangements of viral fusion proteins, and then, the attachment of the host cell by the virus (Figure 8, Supplementary Figure S4 and Table S2). The conjugation of FP with dendrimers could create a steric hindrance and block the S protein in a pre-fusogenic or intermediate conformation, inhibiting a complete and functional interaction between the viral fusion protein and the cell membrane for fusion.

Another important piece of evidence is that the peptide dendrimer has also an antiviral effect on human cells. We tested it on the Calu-3 lung epithelial cell line (Figure 7), showing a very similar antiviral effect with respect to what we observed on Vero-76 cells (Figure 3). Further studies are also mandatory to test the dendrimer’s susceptibility to degradation by proteases and peptidases and to analyze its hemolytic activity. Taken together, these findings indicated that dendrimer R1 can be used as a proof of concept not only to interfere with SARS-CoV-2 infection, but also against other viruses with class I fusion proteins by changing the FP sequence.

## Data Availability

The data presented in this study are available on request from the corresponding author. The authors can confirm that all relevant data are included in the article.

## Supplementary Materials

The following supporting information can be downloaded at: https://www.mdpi.com/article/10.3390/pharmaceutics15122791/s1, Scheme S1: Schematic representation of the peptide dendrimer R1 synthesis process. Figure S1: Chemical structure of Br-acetyl peptide R1 and LC-MS analysis. Figure S2: Chemical structure of branched amino acid core and (B–D) LC-MS analysis. Figure S3: Chemical structure of peptide dendrimer R1 and (B–E) LC-MS analysis. Figure S4: Molecular docking. Table S1: Experimental and theoretical MW of molecules. Table S2. Putative interaction sites occurring between FP (IYKTPPIKDFGGFNFSQIL) and SARS-CoV-2 S protein (PDB 7CYC).
