# Supplementary material for: SARS-CoV-2 Fusion Peptide Conjugated to a Tetravalent Dendrimer Selectively Inhibits Viral Infection
Source: Pharmaceutics. 2023 Dec 17;15(12):2791. doi: 10.3390/pharmaceutics15122791 (PMC10748278; doi:10.3390/pharmaceutics15122791)
Supplement: Supplementary file 1 [file pharmaceutics-15-02791-s001.zip › pharmaceutics-2728944-supplementary.pdf]

pharmaceutics

Supplementary Materials: SARS-CoV-2 Fusion Peptide Conjugated to a Tetravalent Dendrimer Selectively Inhibits Viral Infection

Carla Zannella, Annalisa Chianese, Alessandra Monti, Rosa Giugliano, Maria Vittoria Morone, Francesco Secci, Giuseppina Sanna, Aldo Manzin, Anna De Filippis, Nunzianna Doti and Massimiliano Galdiero

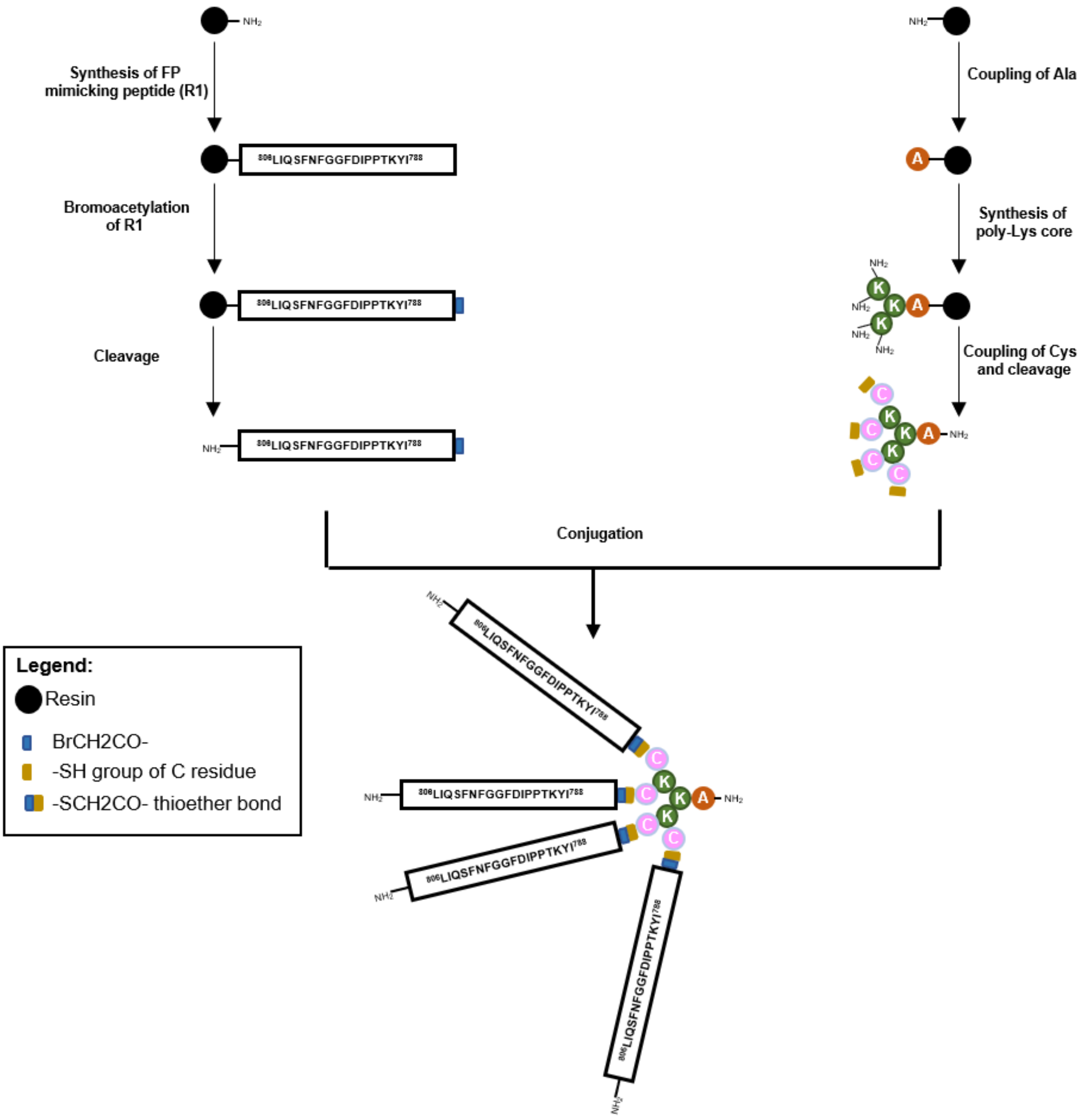

Scheme S1. Schematic representation of the peptide dendrimer R1 synthesis process. A) The FP mimetic peptide (Ile-Tyr-Lys-Thr-Pro-Pro-Ile-Lys-Asp-Phe-Gly-Gly-Phe-Asn-Phe-Ser-Gln-Ile-Leu;) was synthesized as amidated derivative at the C-terminus using the Rink-Amide MBHA resin (loading 0.5 mmol/g) following the N-9-Fluorenylmethyloxycarbonyl (Fmoc) strategy, using standard procedures [1]. The N-terminal bromoacetylation was performed using a 10-fold excess

of bromoacetic acid and DIC as activator (1:1 eq) in DMF for 1 hour at room temperature. (B) Synthetic sequence for the formation of poly-*L*-Lysine dendrimer functionalized in Nα and Nε with Cys residues. After the coupling of Fmoc-*L*-Ala-OH on the resin and the subsequent deprotection of Fmoc, the first Fmoc-*L*-Lys(Fmoc)-OH dissolved in DMF was coupled to the Ala and after removal of the Fmoc groups, two more Fmoc-*L*-Lys(Fmoc)-OH residues were coupled. After a subsequent step of Fmoc deprotection, the Fmoc-*L*-Cys-OH, was coupled followed by a step of Fmoc deprotection. The deprotection steps, the coupling steps and the cleavage from the resin were done using standard procedures [1]. (C) Conjugation reaction. The R1 was conjugated to the four dendrimer's thiol groups according to the organic nucleophilic substitution reaction (S_N2). Amino acids are shown in single code.

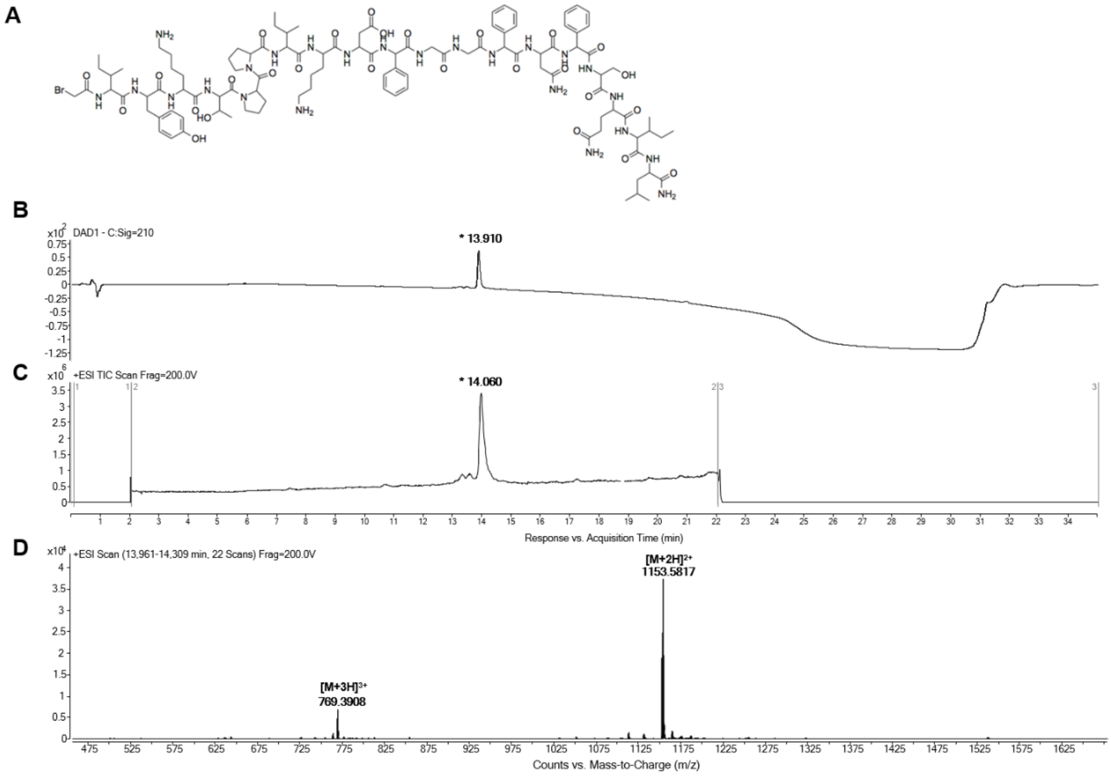

Figure S1. Chemical structure of the Br-acetyl peptide R1 (A) and LC-MS analysis (B-D). The asterisk (*) indicates the chromatographic peak containing the target molecule. The peptide shows a retention time (Rt) of 13,9 min as evidenced of DAD (Diode Array Detector) spectrum. The MS analysis showed the expected mass at m/z: 1153.582 ($[M+2H]^{2+}$) and 769.391 ($[M+3H]^{3+}$). The MS values reported in the figure represent the value of the main peak of the isotopic clusters.

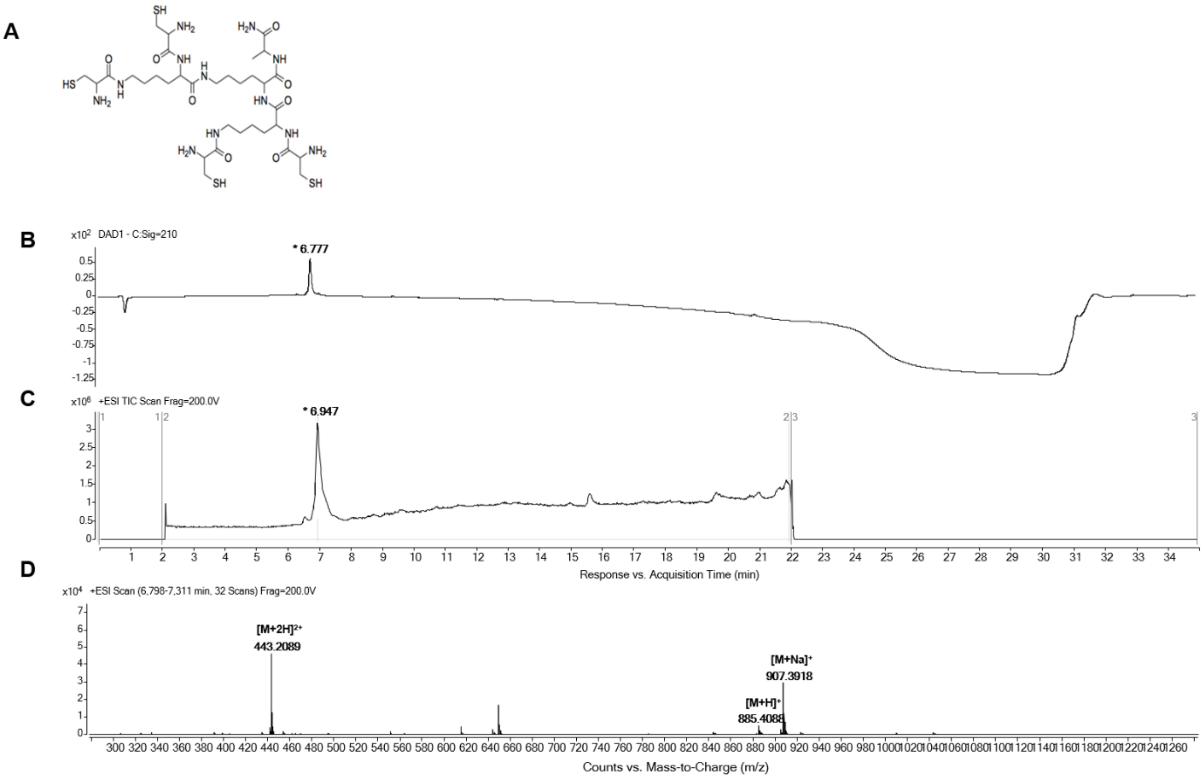

Figure S2. (A) Chemical structure of the branched amino acid core and (B-D) LC-MS analysis. The asterisk (*) indicates the chromatographic peak containing the target molecule. The retention time (tR) value was about 6.78 min as detected by DAD (Diode Array Detector) (B). The MS analysis confirmed the identity of the molecule showing m/z values at: 907.392 ([M+Na]$^+$); 885.401 ([M+H]$^+$) and 443.209 ([M+2H]$^{2+}$). The MS values reported in the figure represent the value of the main peak of the isotopic clusters.

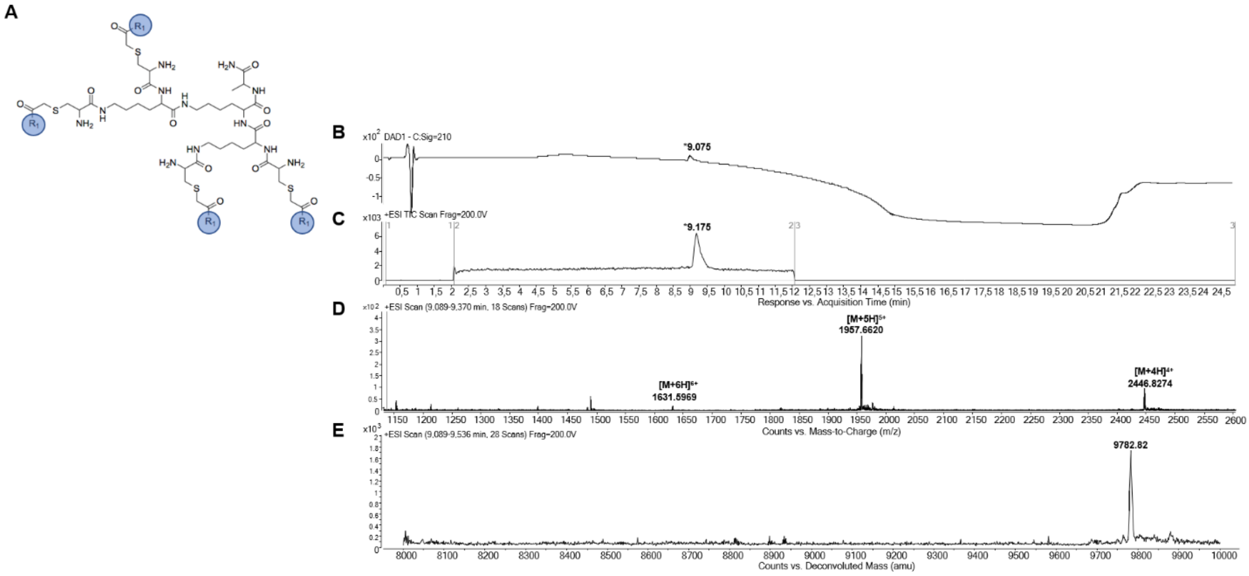

Figure S3. (A) Chemical structure of the peptide dendrimer R1 and (B-E) LC-MS analysis. R1s in the circles indicates the peptide sequences. The asterisk (*) indicates the chromatographic peak containing the target molecule. The retention time (tR) value of the dendrimer was about 9.98 min

as detected by DAD (Diode Array Detector) (B). The MS analysis confirmed the identity of the dendrimer showing m/z values at: 2446.827 ($[M+4H]^{4+}$); 1957.662 ($[M+5H]^{5+}$) and 1631.597 ($[M+6H]^{6+}$). The MS values reported in the figure represent the value of the main peak of the isotopic clusters. The deconvolute mass of 9782.82 amu (D), agrees with the theoretical MW of the molecule.

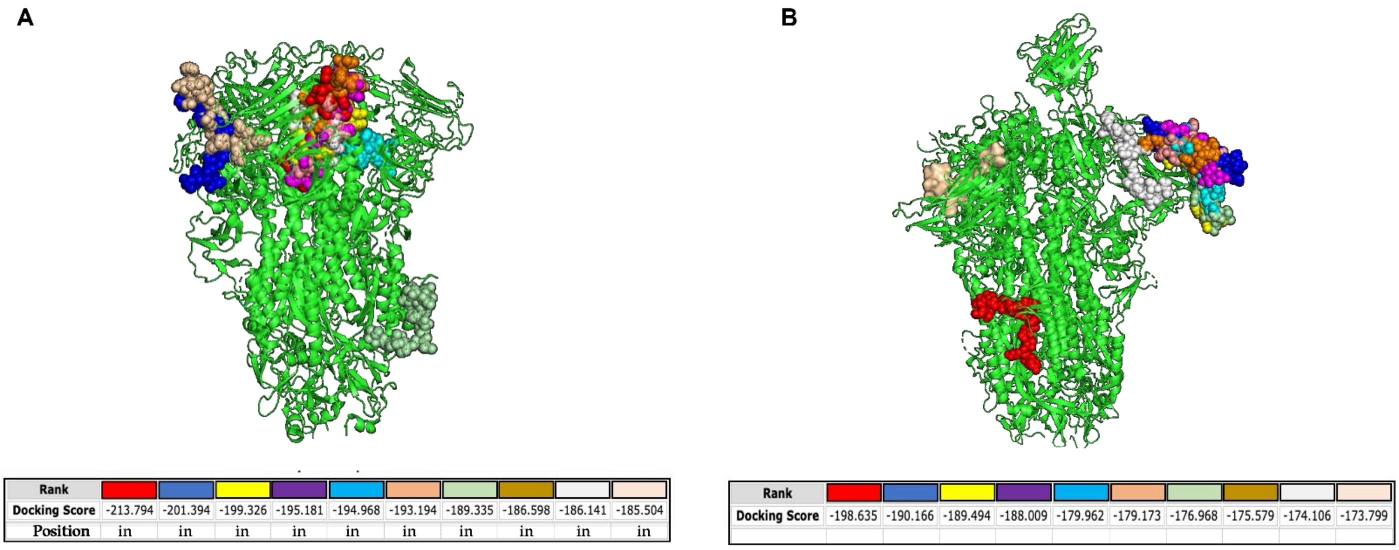

Figure S4. Molecular docking. Molecular prediction of SARS-CoV-2 FP (IYKTPPIK-DFGGFNFSQIL) interacting with (A) HCoV-229E S protein (PDB 7CYC) and (B) HCoV-OC43 S protein (PDB 7SBW) obtained by HPEPDOCK server. The different color code of peptide, represented as balls, refers to the different binding free energy.

Name	MW (theoretical)	MW (experimental)
R1	2305.49	2305.16
Dendrimer	884.37	884.41
Dendrimer R1	9782.33	9782.82

Table S1. Experimental and theoretical MW of molecules tested in this study.

ionic interaction

Ligand atom	Receptor
O11	K417(A) NZ

hydrophobic contact

Ligand atom	Receptor
CA5	F456(A) CD2
C40	F456(A) CE1
C43	F456(A) CZ
CA5	Y473(A) CE2
C40	Y489(A) CD1
C11	Y495(A) CD1
C56	Y380(B) CB
C24	Y380(B) CE2
C79	V382(B) CG1
C45	P384(B) CG
C85	L390(B) CD1
C82	L390(B) CD2
C24	P412(B) CG

hydrogen bond

Ligand atom	Receptor
O23	T415(A) OG1
O24	D420(A) OD2
O2	Y453(A) OH
O2	S494(A) O
N1	Q498(A) OE1
O10	C379(B) O
N16	G381(B) O
N19	G381(B) O
N18	G381(B) O
O27	S383(B) OG
N10	T385(B) OG1
N4	P412(B) O
O15	R403(A) NH2
O20	S383(B) N
O21	K417(A) NZ
O22	K417(A) NZ
N20	K417(A) NZ
O25	K417(A) NZ
O27	S383(B) OG
O23	T415(A) OG1
O2	Y453(A) OH
O2	Q493(A) NE2

weak hydrogen bond

Ligand atom	Receptor
O26	T385(B) CB
O23	T415(A) CB
O12	K417(A) CE

Table S2. Putative interaction sites occurring between FP (IYKTPPIKDFGGFNFSQIL) and SARS-CoV-2 S protein (PDB 7CYC).

References

1. Caporale, A.; Doti, N.; Monti, A.; Sandomenico, A.; Ruvo, M. Automatic procedures for the synthesis of difficult peptides using oxyma as activating reagent: A comparative study on the use of bases and on different deprotection and agitation condi-tions. *Peptides* **2018**, *102*, 38–46
